# Supercritical Phase Inversion to Produce Photocatalytic Active PVDF-coHFP_TiO_2_ Composites for the Degradation of Sudan Blue II Dye

**DOI:** 10.3390/ma15248894

**Published:** 2022-12-13

**Authors:** Mariangela Guastaferro, Lucia Baldino, Vincenzo Vaiano, Stefano Cardea, Ernesto Reverchon

**Affiliations:** 1 Department of Industrial Engineering, University of Salerno, Via Giovanni Paolo II, 132, Fisciano, 84084 Salerno, Italy; 2 C.U.G.RI., InterUniversity Research Center for the Prediction and Prevention of Major Hazards, University of Salerno, Via Giovanni Paolo II, 132, Fisciano, 84084 Salerno, Italy

**Keywords:** TiO_2_ nanoparticles, poly(vinylidene fluoride-co-hexafluoropropylene) membranes, Sudan Blue II dye, supercritical CO_2_ assisted phase inversion, photocatalysis

## Abstract

TiO_2_-loaded poly(vinylidene fluoride-co-hexafluoropropylene) (PVDF-coHFP) membranes were produced by supercritical CO_2_-assisted phase inversion. Three different TiO_2_ loadings were tested: 10, 20, and 30 wt% with respect to the polymer. Increasing the TiO_2_ amount from 10 wt% to 20 wt% in the starting solution, the transition from leafy-like to leafy-cellular morphology was observed in the section of the membrane. When 30 wt% TiO_2_ was used, the entire membrane section showed agglomerates of TiO_2_ nanoparticles. These polymeric membranes were tested to remove Sudan Blue II (SB) dye from aqueous solutions. The adsorption/photocatalytic processes revealed that membrane morphology and TiO_2_ cluster size were the parameters that mainly affected the dye removal efficiency. Moreover, after five cycles of exposure of these membranes to UV light, SB removal was higher than 85%.

## 1. Introduction

Because of their low cost and wide availability, dyes are used as coloring agents in several industries, from food to textiles. For example, Sudan dyes (Sudan I, Sudan II, Sudan III, and Sudan IV) are frequently used in plastics, waxes, oil paint, and printing [1,2]; they are also added as color additives in chili powder and ketchup sauce [3,4]. However, Sudan dyes contain azo-functional groups and aromatic rings; therefore, they can negatively affect human health and produce environmental pollution [5,6]. For this reason, these dyes are banned in most countries; e.g., European Union (EU) directives (Decision 2003/460/EC) restricted the use of Sudan I dyes in all hot chili or related products. This decision has been further extended (2004/92/EC) to the other Sudan dyes (Sudan II, III, and IV). Similar legislation can also be found in other countries [7]. In particular, Sudan Blue II (SB) has a carcinogenic effect and can cause irritation to the skin, eyes, and respiratory tract [8]; consequently, this toxicity creates the need to develop new methods for its detection and removal. Literature analysis shows that several adsorbing materials have been proposed for the removal of Sudan dyes, such as active clay grafted with a silylating agent [9], amine-functionalized magnetic silica nanoparticles [10], magnetic Fe_3_O_4_ on carbon nanotubes [11]. Despite solid-state adsorbents with tuneable selectivity providing a straightforward method to isolate organic pollutants, allowing semi-continuous operations and in-situ recovery [9,10,11,12,13], all the proposed systems suffer a drawback. Only the transfer of the contaminant from one phase to another is obtained, without having the capability to promote oxidation of the organic dyes into not harmful compounds.

As an alternative to adsorption technologies, the advanced oxidation processes (AOPs) have been demonstrated to be reliable for wastewater treatment because they are eco-friendly, economical, and have a high capacity to oxidize several organic pollutants [14,15,16]: heterogeneous photocatalysis is carried out under irradiation of light at room temperature and ambient pressure [17,18,19]. Its effectiveness mainly depends on the band gap, surface area, and recombination rate phenomena of the electron-hole pair of the used semiconductor [20]. Promising photocatalysts are generally those based on titania (TiO_2_) nanoparticles [21,22,23].

Photocatalysis can be coupled with the adsorption process to degrade pollutant molecules transferred from the liquid medium into the material framework, as shown in several literature papers dealing with the removal of dyes (methylene blue [24,25], malachite green [26], rhodamine B [26] and Congo red [27]). This goal was achieved by coupling TiO_2_ or ZnO-based photocatalysts with different organic/inorganic supports in powder form [27]. Therefore, the previous literature analysis suggests that it is possible to use the combination of adsorption with a photocatalytic process to obtain superior results compared with the adsorption-based process alone in terms of removal efficiency, adsorption capacity, and reusability. However, the major drawback is the catalyst removal from water; in particular, when catalysts are used as fine particles, they cannot be removed by gravity settling [28,29]. Consequently, post-treatments are required, such as coagulation that can lead to sludge production and membrane separation that can increase the treatment cost [30].

For this reason, the immobilization of the catalysts on polymeric membranes seems a good alternative compared with other separation processes regarding reduced energy costs, material recovery, and reduction of the environmental impact. However, one of the major drawbacks related to the separation process using catalyst-loaded membranes is represented by the fouling that can lead to a reduced adsorption efficiency due to the accumulation of particles and bacteria on the membrane surface [31,32,33]. Various authors [34,35] discovered that the addition of photocatalyst nanoparticles to the membrane could improve its fouling resistance upon the irradiation of UV light. Therefore, the combination of membrane separation with photocatalysis can represent an efficient, low cost and environmentally friendly technology since it can completely degrade organic dyes and avoid sludge production.

Polymeric membranes show several advantages over inorganic ones, such as an easy pore-forming mechanism, low cost, and high flexibility [36]. Poly (vinylidene fluoride-co-hexafluoropropylene) (PVDF-coHFP) is a copolymer of poly (vinylidene fluoride) (PVDF), widely used as a membrane material due to its high mechanical, chemical, and UV stability [37]. Moreover, the PVDF polymer family allows the production of materials with different morphologies (e.g., thin films, porous membranes, and fiber mats) that may also influence their photocatalytic efficiency [38]. Yadav et al. [39] prepared porous TiO_2_ sheets loaded PVDF-coHFP membranes by a non-solvent induced phase separation (NIPS) process. These membranes were tested for the treatment of textile effluents using two dyes: Congo red 100 ppm and methylene blue 100 ppm. After 24 h of direct contact membrane distillation (DCMD), the positively charged dye molecules were responsible for fouling; after 4 h of UV-light irradiation, all the fouling was removed. Galiano et al. [40] tried to enhance the stability of TiO_2_ dispersion and to reduce particles agglomeration by chemical modification of PVDF-TiO_2_ hollow fibers with the addition of a surfactant: i.e., sodium dodecyl sulfate (SDS). These membranes showed a 97% MB degradation after 5 h of exposure to UV-light irradiation.

However, PVDF-coHFP membranes containing TiO_2_ are generally produced using processes incompatible with green chemistry principles. Kadiev et al. [41] prepared membranes of PVDF-coHFP loaded with TiO_2_ by using electrospinning; however, this process suffers from various drawbacks, such as very low flow rates (≈20 μL/min) and long treatments are required to eliminate the solvent (DMSO) used during the process. Moreover, during the drying step, the catalyst’s loss can also occur due to the lack of interactions or poor adhesion between inorganic TiO_2_ and organic fibers [42,43]. Therefore, the hydrothermal post-treatment is reported as the most suitable technique for inducing in situ TiO_2_ growth after polymeric fiber formation [42,43]. TiO_2_/PVDF catalytic membranes were also prepared by Erusappan et al. [44] using the NIPS process combined with solvent casting. This process is characterized by a long-time formation and a reduced possibility to modulate cell, pore size, and membrane morphology.

Moreover, the membranes produced were also substantially asymmetric. On the other hand, according to the study by Cardea et al. [45] and Reverchon and Cardea [46], the use of supercritical carbon dioxide (SC-CO_2_) assisted phase inversion allowed an enhanced process flexibility, leading to the production of membranes characterized by a regular morphology. Moreover, this process enables the production of composite material in one step and in a short time, it does not require additional post-treatments, and the solvent recovery downstream of the process is easily obtained by depressurization of the system [45,46]. Using SC-CO_2_ assisted phase inversion, PVDF-coHFP membranes with tuneable morphologies were produced: cellular structure, bicontinuous structure, or leafy-like sub-morphology were obtained by changing the polymer concentration [45,46].

Therefore, the aim of this work is to produce, for the first time, PVDF-coHFP membranes loaded with TiO_2_ nanoparticles by SC-CO_2_ -assisted phase inversion to remove a toxic and hydrophobic SB dye from an aqueous solution. To the best of our knowledge, it is worth noting that no papers dealing with photocatalytic membranes applied to the removal of SB dye are present in the literature. The experiments will be carried out to find the loading of TiO_2_ nanoparticles that can guarantee the best catalyst dispersion in the polymeric support, the best adsorption-photocatalytic efficiency of the active membranes, and their reusability.

## 2. Materials and Methods

### 2.1. Materials

Poly (vinylidene fluoride-co-hexafluoropropylene) (PVDF-coHFP, M_n_ = 130,000 Da), acetone (purity > 99.5%), and Sudan Blue II (SB) were bought from Sigma-Aldrich (Milan, Italy). Commercial TiO_2_ nanoparticles (PC 500) were provided by Millenium Inorganic Chemicals (Stallingborough, UK). An SEM image of these TiO_2_ nanoparticles is reported in (Supplementary Material Figure S1). CO_2_ (99.9% purity) was supplied by Morlando Group Srl (Sant’Antimo, Naples, Italy).

### 2.2. Membranes Preparation

PVDF-coHFP-based membranes were prepared by solubilizating 15 wt% polymer in 10 mL of acetone. TiO_2_ nanoparticles, at different concentrations by weight (10%, 20%, and 30%) with respect to PVDF-coHFP, were suspended in the polymeric solution at 50 °C and stirred at 100 rpm. The abbreviations for each prepared membrane are shown in Table 1.

SC-CO_2_ assisted phase inversion (SPI) was performed using a laboratory plant consisting of a 316 stainless-steel cylindrical high-pressure vessel with an internal volume of 200 mL, equipped with a high-pressure pump (mod. LDB1, Lewa, Leonberg, Germany) used to deliver liquid CO_2_. Pressure in the vessel was measured using a test gauge (mod. MP1, OMET, Lecco, Italy) and regulated using a micrometering valve (mod. 1335G4Y, Hoke, Spartanburg, SC, USA). The temperature was controlled using PID controllers (mod. 305, Watlow, Corsico, Milan, Italy). At the exit of the vessel, a rotameter (mod. D6, ASA, Sesto San Giovanni, Milan, Italy) was used to measure the CO_2_ flow rate.

### 2.3. Membranes Characterizations

TiO_2_ particle size in acetone suspension was measured by a dynamic light scattering (DLS) instrument (Zetasizer Nano S, Worcestershire, UK).

Membrane samples were cryo-fractured using liquid nitrogen (SOL, Milan, Italy); then, they were sputter coated with gold (Agar Auto Sputter Coater mod. 108 A, Stansted, UK) at 30 mA for 120 s and analyzed using a field emission scanning electron microscope (FE-SEM, mod. LEO 1525, Carl Zeiss SMT AG, Oberkochen, Germany) to study their morphology.

Brunauer–Emmett–Teller (BET) specific surface area of 15PVDF-coHFP and photocatalytic active membranes (15PVDF-coHFP_10TiO_2_, 15PVDF-coHFP_20TiO_2_, 15PVDF-coHFP_30TiO_2_) was measured by dynamic N_2_ adsorption experiments performed at −196 °C, using a sorptometer 1042 Instrument (Costech, Pioltello, Milan, Italy). Before N_2_ adsorption measurement, the samples were pre-treated at 100 °C for 30 min in a He flow.

To investigate the distribution of TiO_2_ in the polymeric support, an energy-dispersive X-ray spectroscopy (EDX, mod. INCA Energy 350, Oxford Instruments) analysis was performed.

To measure the effective TiO_2_ loading in the polymeric membranes, thermal gravimetric analysis (TGA) was carried out in air (flow rate 100 Ncc/min) using a Mettler TGA/SDTA 851 (Columbus, OH, USA) thermal analyzer and the residual weight percentage at 800 °C was evaluated.

The Raman spectrum of each membrane was recorded using a Dispersive MicroRaman system (InviaRenishaw, Turin, Italy) equipped with a 514 nm diode-laser in the range 100–1000 cm^−1^ Raman shift.

Diffuse reflectance spectra (UV-Vis DRS) were obtained using a Perkin-Elmer spectrophotometer Lambda 35 (Labsphere Inc., North Sutton, NH, USA). All spectra were acquired using an 8° sample-positioning holder, giving a total reflectance relative to a calibrated standard SRS-010-99 (Labsphere Inc., North Sutton, NH, USA).

Fourier transform infrared (FT-IR) spectra were obtained using an M2000 FT-IR spectrophotometer (MIDAC Co, Costa Mesa, CA, USA) to investigate the chemical interactions between SB and the polymeric matrix during the adsorption process. Pellets were prepared by mixing the produced materials and KBr (1:100 by weight). Scans were performed at a resolution of 32 cm^−1^.

### 2.4. Adsorption and Photocatalytic Activity Tests

Adsorption and photocatalytic tests were carried out using an SB initial concentration of 50 mg/L and 50 mL of an aqueous solution. TiO_2_ loaded membrane amount was equal to 0.26 g. A pyrex cylindrical photoreactor, having an internal diameter of 2.6 cm and a height of 9 cm, was used to perform the experiments. The photoreactor was irradiated by two UV lamps (Philips, nominal power: 8 W each and main emission peak at 365 nm). The position of light sources was 15 cm above the upper surface of the batch reactor.

Moreover, the photoreactor was covered with reflecting aluminum foils to ensure that only its upper surface was irradiated. The overall reaction time was 180 min in dark and light conditions. The residual SB concentration was analyzed by recording its absorption at 675 nm using a UV-Vis spectrophotometer (Evolution 201, Thermo Scientific Fischer, Milan, Italy).

## 3. Results

### 3.1. Investigation of PVDF-coHFP Membranes Morphology

15PVDF-coHFP membranes were produced using the SPI process, working at 200 bar and 35 °C. These process conditions were optimized in previous work [46], in which a nanoporous morphology of PVDF-coHFP membranes was obtained.

**Figure 1:**
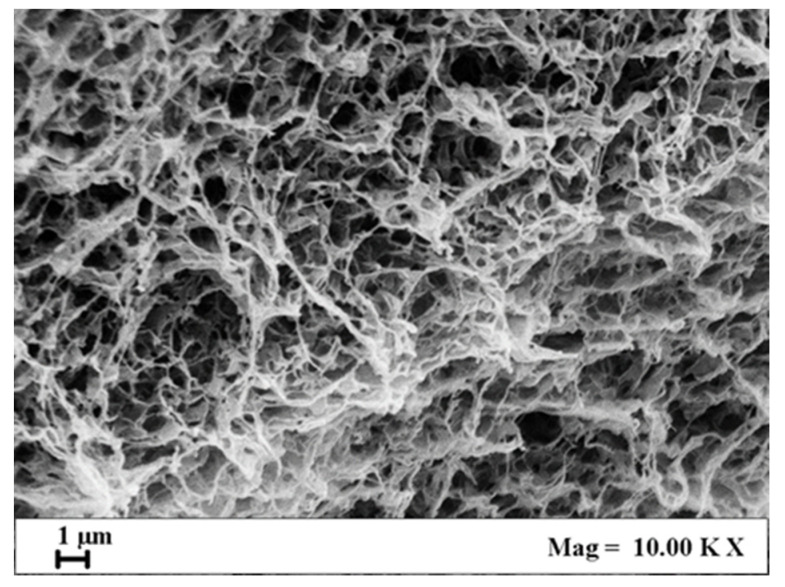
SEM image of 15PVDF-coHFP membrane section.

The bicontinuous structure with a leafy-like morphology was successfully replicated in this work, as can be observed in Figure 1, in which a SEM image of the 15PVDF-coHFP membrane section is reported.

This morphology can be explained considering the kinetic of cell formation that was mainly affected by a solid-liquid demixing process. This phenomenon generally occurs in the crystallizable segments of semi-crystalline polymers, leading to membranes consisting of interlinked crystalline particles [46].

**Figure 2:**
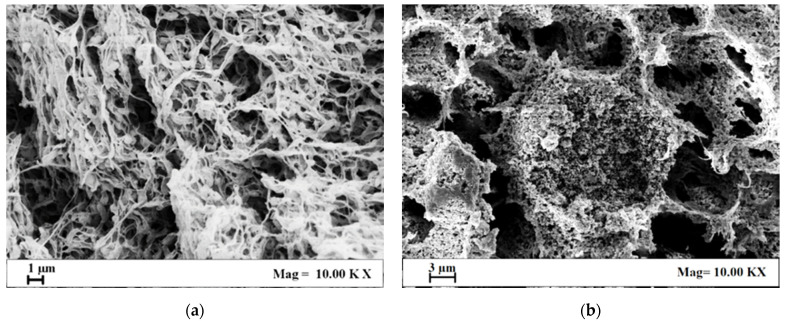

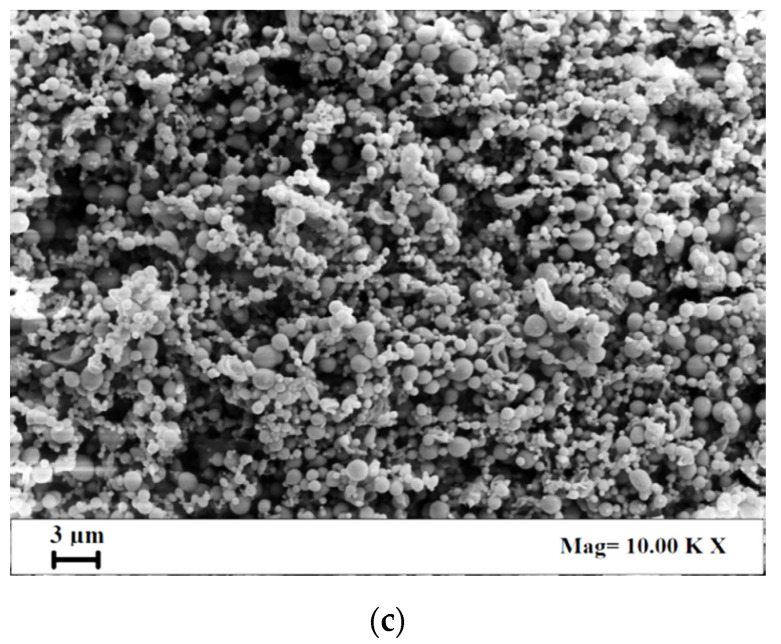
SEM images of 15PVDF-coHFP_10TiO_2_ (**a**), 15PVDF-coHFP_20TiO_2_ (**b**), and 15PVDF-coHFP_30TiO_2_ (**c**).

Then, different TiO_2_ nanoparticle loadings were attempted: 15PVDF-coHFP membranes were loaded with 10 wt%, 20 wt%, and 30 wt% TiO_2_ with respect to the polymer amount. SEM images related to these loaded membranes are reported in Figure 2.

A TiO_2_ loading of 10 wt% did not significantly modify the polymeric membrane morphology. In Figure 2a, it is possible to identify a leafy-like morphology characterized by a low presence of TiO_2_ nanoparticles along the filaments. A TiO_2_ loading equal to 20 wt%, instead, led to an increase in the starting solution viscosity and, as a result, the pathway in the ternary diagram [46] moved to the pure polymer apex (between spinodal and binodal curves), leading to a cellular structure characterized by nanoporous walls and well-dispersed nanometric titania (Figure 2b). Agglomeration was obtained when a TiO_2_ loading of 30 wt% was added to the polymeric solution, and clusters of nanoparticles were formed (Figure 2c).

The presence of TiO_2_ nanoparticles at different percentages in the membranes did not interfere with BET surface area values that refer to the mesoporosity evaluation of the samples. They were equal to almost 35 m^2^/g for all produced membranes.

**Figure 3:**
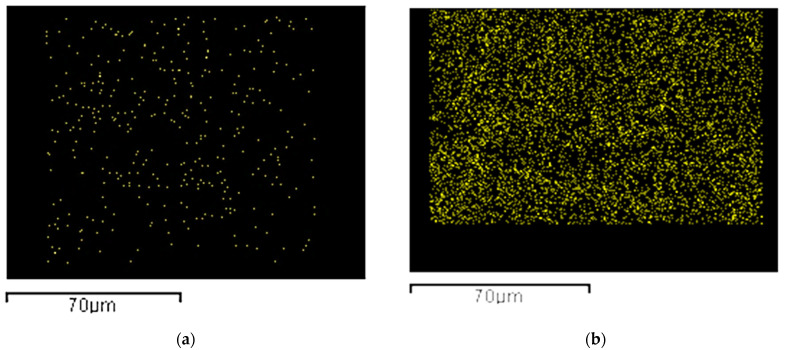

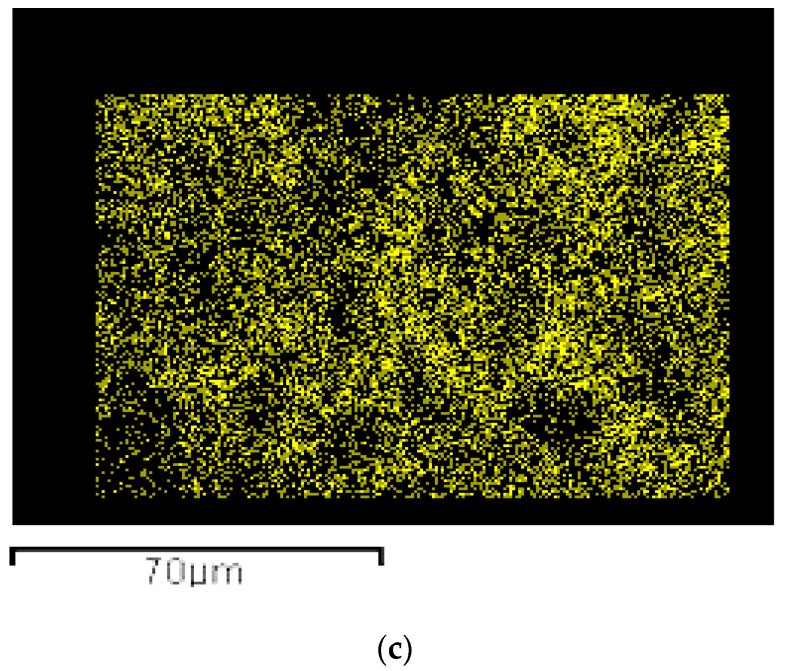
EDX Ti elemental mapping related to 15PVDF-coHFP_10TiO_2_ (**a**), 15PVDF-coHFP_20TiO_2_ (**b**), and 15PVDF-coHFP_30TiO_2_ (**c**).

These results were also confirmed by the elemental analysis obtained from EDX maps (Figure 3) that provides Ti distribution in the membrane section. In Figure 3a, it is possible to identify only a few amounts of Ti, whereas a uniform dispersion of Ti element in the entire section can be detected in Figure 3b. Clusters of micrometric dimensions can be observed in Figure 3c.

**Figure 4:**
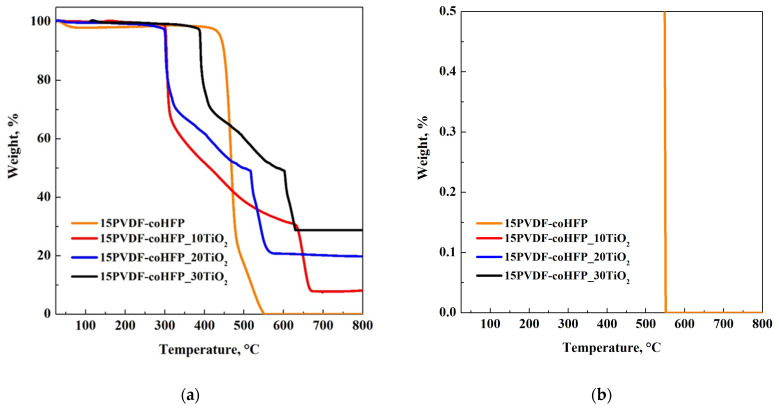
(**a**) TGA analyses of 15PVDF-coHFP (orange curve), 15PVDF-coHFP_10TiO_2_ (red curve), 15PVDF-coHFP_20TiO_2_ (blue curve), 15PVDF-coHFP_30TiO_2_ (black curve); (**b**) enlargement of the orange curve in the range 0–0.5%.

The effective loadings of TiO_2_ into polymeric membranes were evaluated considering the residual weight percentage from TGA analyses at 800 °C. TGA curves and the enlargement of pure polymer curve from 0 to 0.5% are reported in Figure 4a,b, accompanied by Table 2, summarizing the TiO_2_ content in the samples.

TGA analysis was carried out in the air, allowing the total oxidation of organic carbon contained in the sample. As can be observed in Figure 4b, the decomposition of pure PVDF-coHFP (orange curve) was complete in the range of the investigated temperature. Therefore, it is possible to claim that, also in the composite materials, the complete oxidation of polymeric fraction occurred. Consequently, the final residue value was only related to TiO_2,_ and its value (±1%), reported in Table 2, corresponded to the loading used in the preparation step.

### 3.2. Raman Spectroscopy and UV-Vis DRS Results

**Figure 5:**
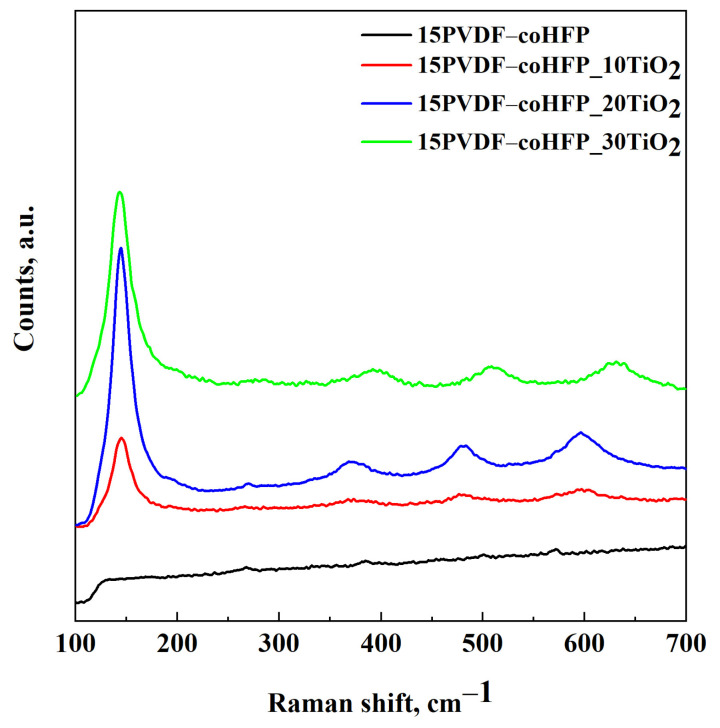
Raman spectroscopy results of unloaded and loaded 15PVDF-coHFP membranes.

Raman spectroscopy was performed on the composite membranes to investigate possible modifications in the TiO_2_ structure after supercritical processing. The results are reported in Figure 5.

This analysis showed the presence of the polymer (black line) and the active compound (TiO_2_) in the final composite materials (red line, blue line, and green line in Figure 5). PVDF-coHFP (black line) showed signals at very low intensity, and for this reason, its enlargement is reported in Figure S2; according to the study reported by Pavlovic et al. [47], the presence of signals in the range from 780 to 900 cm^−1^ revealed that the crystallization of the copolymer is related to the formation of α, β and γ, crystals. When the polymeric blend was produced, the four characteristic peaks of TiO_2_ were also identified; i.e., Raman spectra of PVDF-coHFP_TiO_2_ membranes displayed the phonon modes associated with anatase Eg(1), B1g(1), A1g(1) + B1g(2) and Eg(3) at 144, 399, 520 and 640 cm^−1^, respectively, that correspond to the four modes of TiO_2_ in the 110–700 cm^−1^ range [48].

Before performing photocatalytic tests, UV-Vis reflectance spectra of the solid matrix were carried out (Figure S3). The spectrum of the PVDF-coHFP membrane showed no absorbance peaks, whereas an overall more substantial absorbance at around 350 nm can be observed for the PVDF-HFP_TiO_2_ spectrum. Therefore, during the photocatalysis experiments, the polymeric matrix will not absorb the UV radiation that will be wholly used to activate TiO_2_ particles embedded into the membranes.

### 3.3. Photocatalytic Activity of PVDF-coHFP Membranes Loaded with TiO_2_

**Figure 6:**
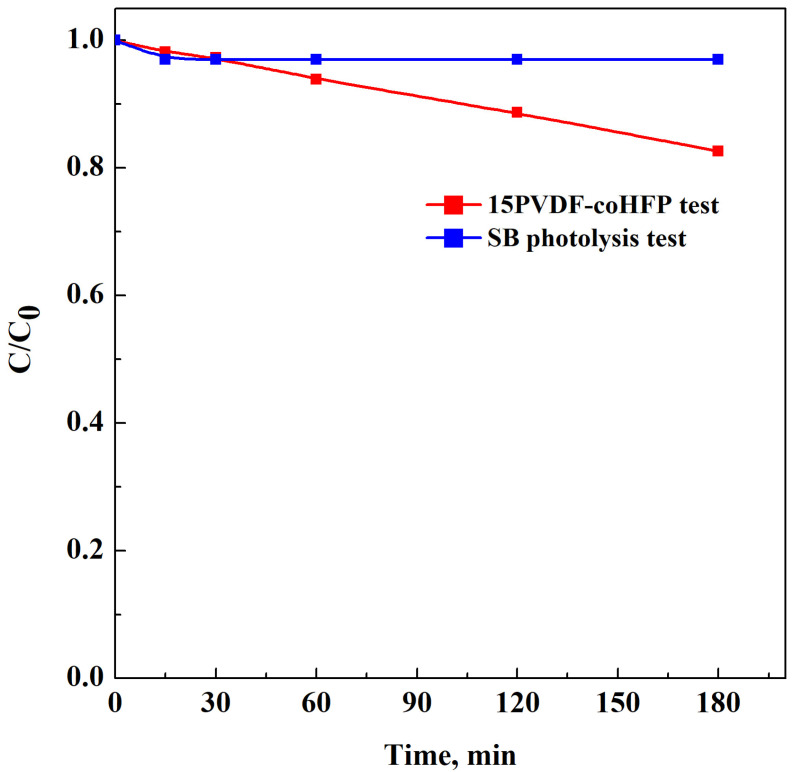
SB removal using 15PVDF-coHFP membrane under UV light (red line) and SB photolysis test (blue line).

PVDF-coHFP membrane (0.26 g) was immersed in 50 ppm of SB aqueous solution. After 2 h of UV irradiation, only a slight reduction of dye concentration was recorded, as shown in Figure 6 (red line).

Degradation of SB was also preliminarily studied in the absence of the photocatalytic active membrane under exposure to UV-light irradiation (i.e., a photolysis test). Figure 6 (blue line) shows the behavior related to this test. It indicates that the presence of UV light only induced a slight decrease in the SB relative concentration, underlining the robust stability of SB.

**Figure 7:**
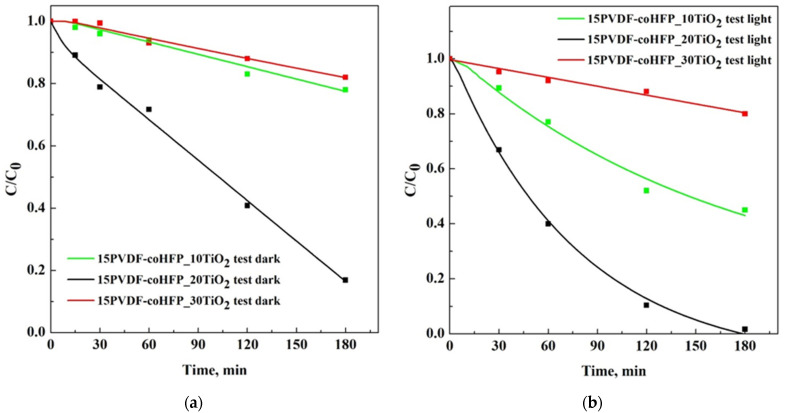
Comparison among 15PVDF-coHFP_10TiO_2_, 15PVDF-coHFP_20TiO_2_, and 15PVDF-coHFP_30TiO_2_ for the degradation of SB in dark conditions (**a**) and under the exposure to UV light (**b**).

Figure 7 reports the results of SB degradation using PVDF-coHFP loaded membranes, achieved in dark conditions (Figure 7a) and under light exposure (Figure 7b).

When the tests were carried out without UV light (Figure 7a), the amount of adsorbed dye was mainly due to the membrane morphology: 15PVDF-coHFP_10TiO_2_ and 15PVDF-coHFP_30TiO_2_ membranes led to 20% SB removal; whereas, 15PVDF-coHFP_20TiO_2_ led to 80% SB removal. This result can be explained considering that 15PVDF-coHFP_20TiO_2_ membranes were characterized by a leafy-cellular structure, and good dispersion of nanometric TiO_2_ particles was achieved (Figure 2b). In particular, the leafy-cellular structure of the membranes was characterized by higher macroporosity values compared with the other morphologies shown in Figure 2a,c. Consequently, a more efficient SB adsorption process can be obtained. Moreover, improved dispersion of TiO_2_ nanoparticles possibly favored a higher hydrophilic behavior of the PVDF-coHFP matrix, improving the contact between SB aqueous solution and PVDF-coHFP membrane, similar to what was observed in the literature [49].

**Figure 8:**
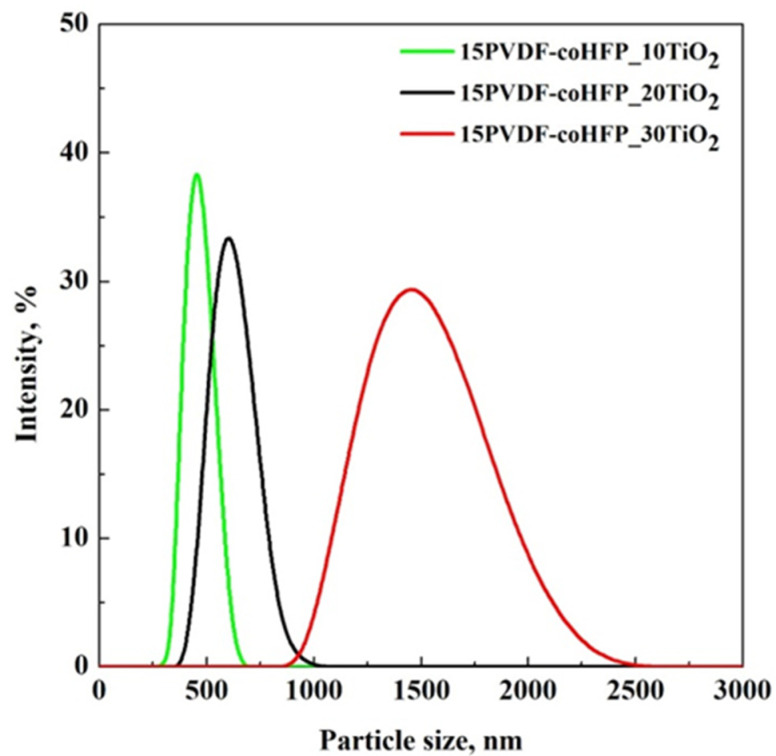
DLS results of the starting PVDF-coHFP solutions loaded with TiO_2_.

The experiments performed in the presence of UV light (Figure 7b) showed more significant dye removal kinetics for each tested membrane. These results can be explained considering that TiO_2_ was activated by UV exposure and, consequently, it was able to degrade the adsorbed pollutant, making available the active site to adsorb the organic dye molecules still present in the aqueous solution [50]. In the presence of light, the best result was achieved using a 15PVDF-coHFP_20TiO_2_ membrane that produced the complete SB degradation after 180 min (Figure 7b, black line). In dark conditions, the SB removal by only adsorption was almost 85% after the same treatment time (Figure 7a, black line). When the 15PVDF-coHFP_30TiO_2_ membrane was tested, the results obtained under UV light were almost the same obtained in the dark. In the case of 15PVDF-coHFP_30TiO_2_ membranes, the entire macroporosity was occupied by TiO_2_ particles, generating agglomerate size significantly higher than the membrane at 20 wt% of TiO_2_ (as observed from FE-SEM analysis), therefore, inducing a worsening of photocatalytic performances, consistently with the literature dealing with TiO_2_ immobilized on macroscopic supports [51]. DLS analysis reported in Figure 8 confirmed TiO_2_ nanoparticles agglomeration, even in the starting solutions used for the SPI process.

It is possible to observe that the mean size of TiO_2_ clusters in the solutions increased from 460 nm to 615 nm for a TiO_2_wt% equal to 10% and 20%, respectively. However, DLS results overestimated the size of these TiO_2_ particle clusters in the 15PVDF-coHFP solutions since they were not found in the produced membranes, as evidenced by SEM results (Figure 2a,b). It is worth noting that when TiO_2_ content in the polymeric solution increased up to 30 wt%, the size of particle clusters dramatically increased to about 1500 nm, in agreement with SEM images observation (Figure 2c). Since for a photocatalyst content up to 20 wt%, the TiO_2_ cluster size was smaller than that one obtained at 30 wt% TiO_2_ loading, it is possible to argue that the effect of photocatalyst cluster size strongly affected both adsorption properties and photocatalytic activity of the membranes (see Figure 7a,b).

### 3.4. Reusability Test of 15PVDF-coHFP_20TiO_2_ Membrane

**Figure 9:**
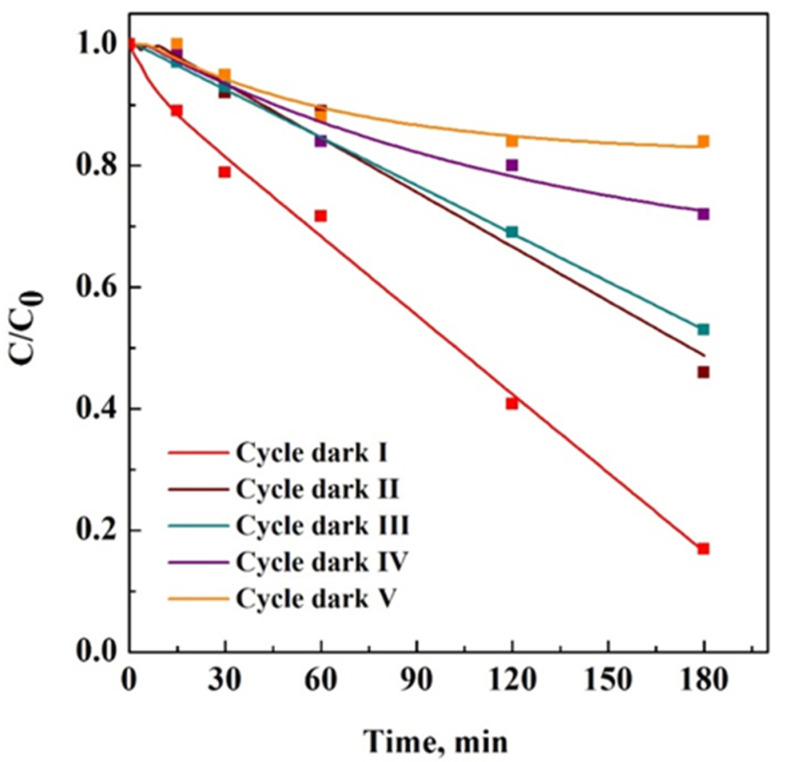
Adsorption cycles in dark conditions of 15VPDF-coHFP_20TiO_2_ membrane.

A reusability test was performed after drying at room temperature of the 15PVDF-coHFP_20TiO_2_ membrane recovered from the treated aqueous solution at the end of the adsorption/photocatalytic process and reused without regeneration step among the different cycles. Figure 9 reports five cycles of SB adsorption in dark conditions on the same 15PVDF-coHFP_20TiO_2_ membrane.

When the first cycle was carried out, the surface of the photocatalytic membrane was free from adsorbed SB dye molecules, and its removal was equal to 80% within the first 180 min. During the fifth reuse cycle, a more significant number of molecules remained adsorbed on the membrane surface. Therefore, the adsorption capacity decreased to 12%, evidencing that the adsorption property of the tested membrane was almost wholly lost.

**Figure 10:**
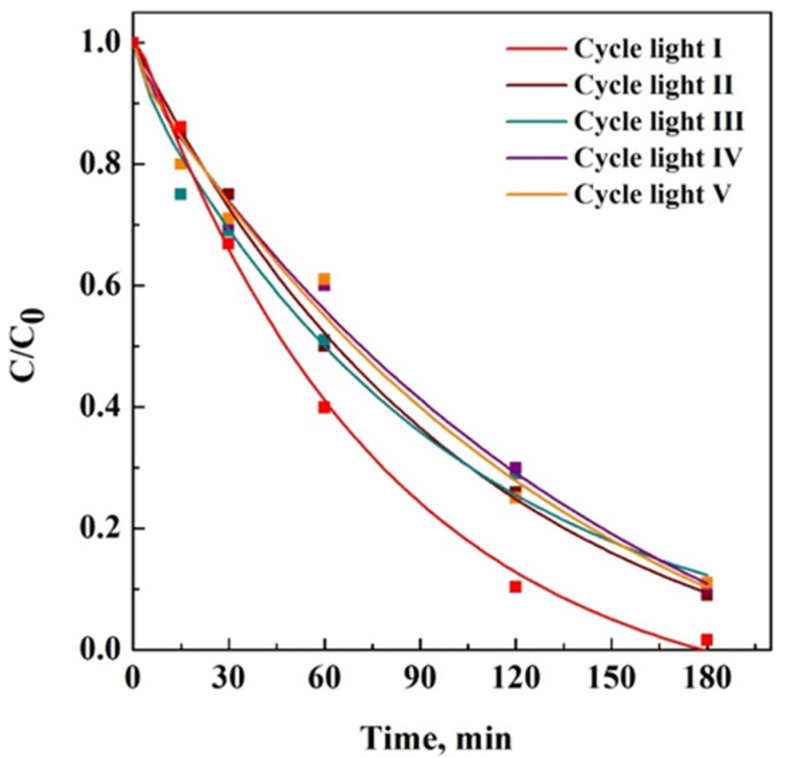
Adsorption cycles of 15VPDF-coHFP_20TiO_2_ membrane under exposure to UV light.

On the contrary, after five consecutive cycles in the presence of UV light, SB removal in all cases was more significant than 85% after 180 min of treatment, as reported in Figure 10. This result demonstrated that photocatalytic oxidation could degrade the adsorbed dye molecules in a one-step treatment, preserving the membrane removal capacity.

**Figure 11:**
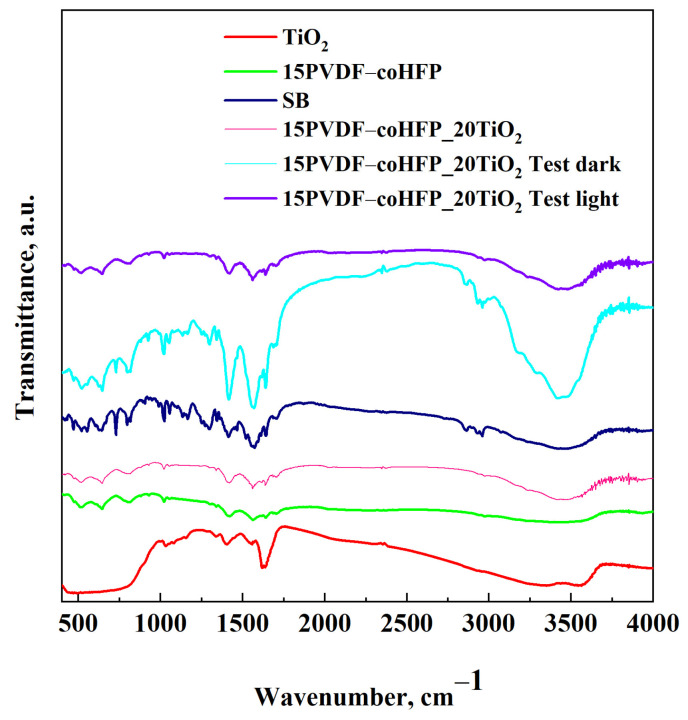
FT-IR spectra of TiO_2_ (red line), PVDF-coHFP (green line), SB (blue line), 15PVDF-coHFP_20TiO_2_ (pink line), 15PVDF-coHFP_20TiO_2_ after cycle V in dark conditions (cyan curve) and 15PVDF-coHFP_20TiO_2_ after cycle V under UV-light exposure (violet curve).

This result was confirmed by FT-IR spectra reported in Figure 11.

FT-IR spectrum of PVDF-coHFP showed bands at 2900 cm^−1^ (C-H stretching), 1049 cm^−1^ (CH_2_ wagging), 1336 cm^−1^ (antisymmetric -CF_2_ stretch), 1020 cm^−1^ (-CF_3_ out-of-plane deformation), 927 and 644 cm^−1^ (related to α-phase), 813 cm^−1^ (related to β-phase) and at 873 cm^−1^ (amorphous phase) [52]. TiO_2_ showed a broad band in the range between 3000 and 3600 cm^−1^ that was related to -OH stretching vibrations of adsorbed water molecules [53]. The signals at about 1630 cm^−1^ could be related to the Ti-OH stretching mode, whereas the bands at 1340–1500 cm^−1^ could be attributed to the carboxyl (C=O). The flexion vibrations of O-Ti-O and the bending vibrations of Ti-O were in the 1000–500 cm^−1^ range [54]. These contributions were all present in the composite membranes (violet and cyan curve). 

The violet curve and the pink curve, which are related to the FT-IR spectrum of the membrane after five cycles of UV irradiation and the composite membrane after the process, presented only the bands related to the membrane and the TiO_2_ nanoparticles (in the range between 3000 cm^−1^ and 3745 cm^−1^). In contrast, the cyan curve (associated with the membrane after the test performed in dark conditions) also showed the presence of the SB characteristic bands (-OH stretching vibrations mode in the range between 3200 and 3600 cm^−1^ and N-C stretching vibrations at 1317 cm^−1^ [55]) reported in the blue curve. This result highlighted that SB in dark conditions was adsorbed on the membrane surface, and the degradation occurred only under the exposure to UV light since all the signals associated with SB were absent in the spectrum of 15PVDF-coHFP_20TiO_2_ membrane collected after five cycles of irradiation.

## 4. Conclusions

TiO_2_-loaded 15PVDF-coHFP membranes were successfully produced by SC-CO_2_ -assisted phase inversion and were tested, for the first time, for SB removal. In particular, the produced membranes were used for SB using a combination of two processes (adsorption/photocatalysis). The adsorption of the 15PVDF-coHFP -based membrane showed poor adsorption of SB, producing only a 20% of dye reduction in the liquid phase. Upon the addition of TiO_2_, two significant phenomena were observed. First, SB adsorption was enhanced, probably due to increased membrane hydrophilicity that allowed a more effective contact between the polymeric matrix and the aqueous solution. Second, in the presence of UV light, the active porous materials also allowed the total dye removal in 180 min, also showing good reusability properties. The photocatalytic tests performed on PVDF-coHFP and PVDF-coHFP_TiO_2_ membranes demonstrated that the photocatalytic efficiency was highly related to the membrane morphology and TiO_2_ particle dispersion. Indeed,15PVDF-coHFP_20TiO_2_ membrane showed the best performance in terms of simultaneous adsorption and photocatalytic degradation since the cellular structure of the composite allowed a better dispersion (confirmed by EDX elemental mapping) and exposure of the catalyst to the UV-light. Overall, the results of the present work can be considered a proof of concept for developing hybrid materials with photocatalytic properties that, under continuous UV-light exposure, can mitigate membrane fouling phenomena, improving the removal of hydrophobic organic compounds and providing an important self-cleaning behavior.

## Figures and Tables

**Table 1:** Name abbreviation of the membranes tested in this work.

Membrane Composition	Membrane Name Abbreviation
15 wt% PVDF-coHFP	15PVDF-coHFP
15PVDF-coHFP + 10 wt% TiO_2_/PVDF-coHFP	15PVDF-coHFP_10TiO_2_
15PVDF-coHFP + 20 wt% TiO_2_/PVDF-coHFP	15PVDF-coHFP_20TiO_2_
15PVDF-coHFP + 30 wt% TiO_2_/PVDF-coHFP	15PVDF-coHFP_30TiO_2_

**Table 2:** Comparison between nominal and measured loading of TiO_2_ in the polymeric membranes.

Nominal Loading of TiO_2_ [wt%]	Measured Loading of TiO_2_ [wt%]
10	8.9
20	19.8
30	28.2

## Data Availability

Not applicable.

## Supplementary Materials

The following supporting information can be downloaded at: https://www.mdpi.com/article/10.3390/ma15248894/s1. Figure S1: SEM image of commercial TiO_2_ (P500). Figure S2: Raman spectrum of 15PVDF-coHFP. Figure S3: UV-vis DRS on PVDF-coHFP and PVDF-coHFP_TiO_2_ membranes.
