# Supplementary material for: Supercritical Phase Inversion to Produce Photocatalytic Active PVDF-coHFP_TiO2 Composites for the Degradation of Sudan Blue II Dye
Source: Materials (Basel). 2022 Dec 13;15(24):8894. doi: 10.3390/ma15248894 (PMC9782530; doi:10.3390/ma15248894)
Supplement: Supplementary file 1 [file materials-15-08894-s001.zip › materials-2045578-supplementary.pdf]

materials

Article

Supercritical Phase Inversion to Produce Photocatalytic Active PVDF-coHFP_TiO_2 Composites for the Degradation of Sudan Blue II Dye

Mariangela Guastaferro [1], Lucia Baldino [1,2,*], Vincenzo Vaiano [1,*], Stefano Cardea [1] and Ernesto Reverchon [1,2]

1 Department of Industrial Engineering, University of Salerno, Via Giovanni Paolo II, 132, Fisciano, 84084 Salerno, Italy
2 C.U.G.RI., InterUniversity Research Center for the Prediction and Prevention of Major Hazards, University of Salerno, Via Giovanni Paolo II, 132, Fisciano, 84084 Salerno, Italy
* Correspondence: lbaldino@unisa.it (L.B.); vvaiano@unisa.it (V.V.)

Supplementary Materials

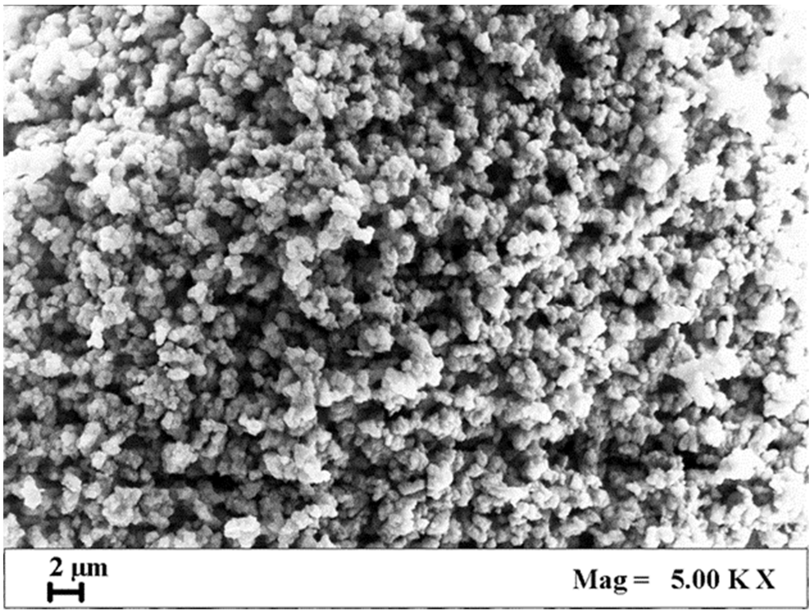

Figure S1. SEM image of commercial TiO_2 (P500).

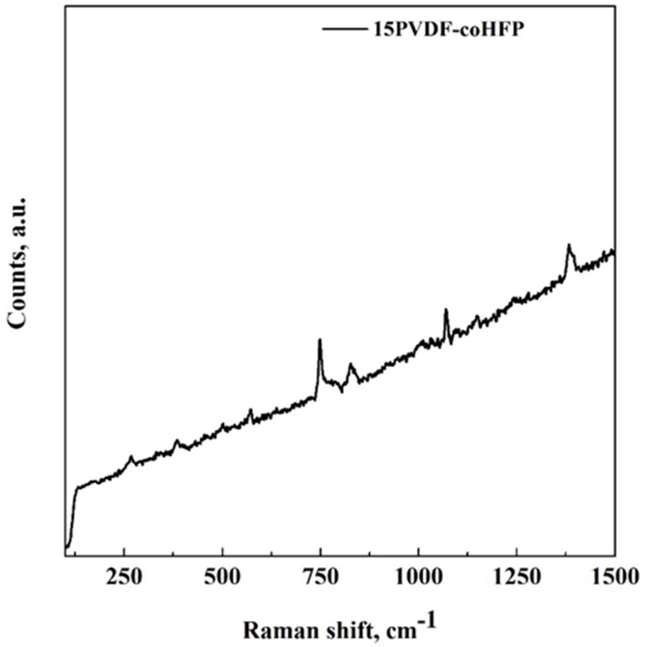

Figure S2. Raman spectrum of 15PVDF-coHFP.

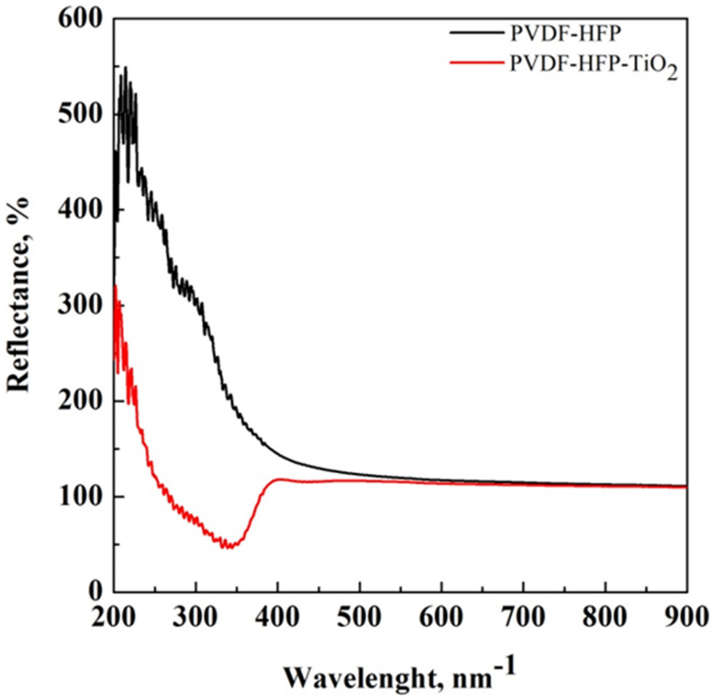

Figure S3. UV-vis DRS on PVDF-coHFP and PVDF-coHFP_TiO2 membranes.
